#  Mechano-Regulation of Alternative Splicing

**DOI:** 10.2174/138920213804999156

**Published:** 2013-03

**Authors:** Huan Liu, Liling Tang

**Affiliations:** Key Laboratory of Biorheological Science and Technology, Ministry of Education, College of Bioengineering, Chongqing University, Chongqing 400044, China

**Keywords:** Mechanical stress, Alternative splicing, Regulation mechanism, Splicing isoform, Ion channel, Signaling pathway.

## Abstract

Alternative splicing contributes to the complexity of proteome by producing multiple mRNAs from a single gene. Affymetrix exon arrays and experiments *in vivo* or *in vitro* demonstrated that alternative splicing was regulated by mechanical stress. Expression of mechano-growth factor (MGF) which is the splicing isoform of insulin-like growth factor 1(IGF-1) and vascular endothelial growth factor (VEGF) splicing variants such as VEGF_121_, VEGF_165, _VEGF_206_, VEGF_189_, VEGF_165_ and VEGF_145 _are regulated by mechanical stress. However, the mechanism of this process is not yet clear. Increasing evidences showed that the possible mechanism is related to Ca^2+ ^signal pathway and phosphorylation signal pathway. This review proposes possible mechanisms of mechanical splicing regulation. This will contribute to the biomechanical study of alternative splicing.

## 1 INTRODUCTION

Pre-mRNA alternative splicing is an essential step in eukaryotic gene expression, because the binding properties, intracellular localization, enzymatic activity, protein stability and posttranslational modifications of most proteins are determined by this process [1]. Splicing is carried out by a multicomponent machinery known as spliceosome, in which a series of biochemical reactions takes place. Spliceosome consists of approximately two hundred proteins and five different small nuclear ribonucleoprotein particles (U1, U2, U4, U5 and U6 snRNPs). Fig. (**1**) describes the stepwise spliceosome assembly pathway [2, 3].

The regulation of alternative splicing is bound up with extracellular environment. Many external stresses, such as heat shock, ultraviolet (UV) irradiation, genotoxic stress or DNA damage change the expression of splicing isoforms. Researches propose at least three possible mechanisms of splicing events induced by extracellular stress: i) stresses affect the assembly of spliceosome by suppressing splicing factor activity; ii) stresses alter splicing factor function; iii) stresses alter splicing factor location.

In addition to all above stresses, alternative splicing is also found to be sensitive to mechanical stimuli. Mechanical stress not only promotes the growth and survival of cells, but also regulates metabolic processes such as gene expression and tissue structure in different kinds of cells [4]. Cells responding to mechanical signals are named mechanocytes, and include endothelial cells, fibroblasts, osteoblasts, smooth muscle cells etc. In those mechanocytes, alternative splicing is found to be controlled by mechanical stress. Alternative splicing of insulin-like growth factor 1(*IGF-1*) was first found in muscle. Expression of splicing forms of* IGF-IEa* and *IGF-IEb *was observed in rodent and *IGF-IEc* in human [5]. Experiments showed that mechanical stretching induced expression of mechano growth factor (*MGF*) which is one of IGF-I splicing isoforms [6]. Similar production of *MGF* was also observed in osteoblasts [7], neurons [8], cardiac muscle cells [9], and tendon [10]. Moreover, genome analysis demonstrated that alternative splicing was regulated by mechanical stress. The potential alternative splicing events induced by mechanical loading in bone were evaluated by Affymetrix exon arrays. In this experiment, the number of alternative genes was 992. These alternative genes belonged to different categories of Gene ontology, such as apoptosis, calcium, cell cycle, cytokine and so on [11]. All of these results showed that mechanical stress regulated alternative splicing events. Unfortunately, the mechanism of this kind of regulation is still unclear.

Evidences are accumulating that alternative splicing plays important roles in proliferation, differentiation and development of cells, and associates with many kinds of human diseases [12, 13]. Recently, the mechanism of alternative splicing has attracted increasing attention. In this paper, we review researches on the regulation of alternative pre-mRNA splicing in response to mechanical stress and propose possible mechanisms of this process. 

## 2 SPLICE VARIANTS OF GENES IN RESPONSE TO MECHANICAL STRESS

It is known that gene expression is regulated by different kinds of mechanical stimulation. Remarkably, many of these genes have splice variants, including vascular endothelial growth factor (*VEGF*), Insulin-like Growth Factor (*IGF-I*), Tension-induced/inhibited proteins (*TIPs*), Tenascin C (*TnC*), collagen XII, Versican, CD44 and Serum response factor (*SRF*). Some splicing variants of these stress sensitive genes are controlled by mechanical stress. These are the direct evidences of alternative splicing in response to mechanical stress.

In many different tissues and cells both normal and diseased, *VEGF* appears as a mechanical stimulation-inducible expression factor and both physical and excessive physical mechanical stress promote its expression [14, 15]. As the result of alternative splicing, there are multiple *VEGF* isoforms, including *VEGF_189_, VEGF_165_, VEGF_121_, VEGF_206_, VEGF_183_, VEGF_145_, VEGF_148_, VEGF_120_, VEGF_164_* and so on [16]. 

IGF-I is related to cell proliferation, differentiation and survival. Its pre-mRNA encodes different protein isoforms by alternative splicing in response to mechanical stimulation, *IGF-IEa*, *IGF-IEb* and *IGF-IEc. IGF-IEb* is also called mechano-growth factor (*MGF*) which is found to be up-regulated in injury skeletal muscle [17]. *MGF* promotes tissue repair and regeneration [18].

TIPs have three isoforms *TIP-1, TIP-2 *and *TIP-3*, which are generated by alternative splicing from a single gene. Sequences of *TIPs* contain motifs encoding nuclear receptor coregulators, chromatin remodeling factors with histone acetyltrnsferase and histone deacetylase [19]. Studies showed that *TIP-1 *was only expressed in undifferentiated lung embryonic mesenchymal cells by stretch. The expression of *TIP3* in progenitor cells was not affected by stretch. However, its expression in smooth muscle cells was suppressed. Further experiment suggested that *TIP-1* and *TIP-3* were involved in early response to stretch. *TIP-2* was also a stretch-sensitive protein, which was expressed in NIH3T3 cells in response to stretch [20].

* TnC* is related to many important cell functions, such as adhesion, migration, development and tumor metastasis [21]. *TnC* splicing isoforms contain the insertion of FnIII domains between constitutive domains FnIII 5 and FnIII 6 [22]. The isoform without any alternative domains and the isoform with FnIII D domain were expressed in porcine TM cells. Mechanical stretching induced a twofold increase of FnIII D domain in TM cells after stretching for 48 hours [23]. 

When exposed to mechanical stretching, a novel collagen XII splice isoform was expressed in TM cells [23].

* Versican* is a member of hyaluronan-binding proteoglycans family. It interacts with ECM and affects cell adhesion, migration, proliferation and ECM assembly [24]. Versican has four different alternative transcripts (*V0, V1, V2* and *V3*) which contain both, either or neither of the two alternative exons (7 and 8). The expression of *V1* is significantly up-regulated by mechanical stretching, however the abundance of *V2* and *V0* remains low without significant changes. 

* CD44* is transmembrane glycoproteins related to cell-cell and cell-matrix interactions [25]. All the *CD44* members are produced by alternative splicing from one gene with different variable exons (v1-v10). Porcine TM cells express variable exons v3, v7 and v8. When subjected to stretching, the proportion of v7 and v8 domains increased at 3-fold after 12 hour stretching and decreased at 1.5-fold after 48 hours stretching [23].

* SRF* regulates the expression of muscle-specific gene. *SRF-M* is one of its isoforms lacking exon5. The other two *SRF *isoforms is *SRF-S* lacking exon4 and exon5, and *SRF-I* lacking exon3, exon4 and exon5 [26]. Mechanical stress modulates *SRF* alternative splicing. Stretching for 4 hours, mechanical stress suppressed the synthesis of *SRF-M* mRNA, while *SRF* mRNA levels were increased. After 12 hours stretching, the result was just opposite [27].

All of these examples showed that mechanical stress could regulate the expression of different genes. Splicing isoforms of these genes expressed differentially in response to extracellular stimulation. Mechanical stress is an important regulatory element of alternative splicing.

## 3 THE MECHANISM ANALYSIS OF ALTERNATIVE SPLICING IN RESPONSE TO MECHANICAL STRESS

### Ca^2+^ Signaling Pathway

Almost every cellular process is directly or indirectly related to Ca^2+ ^signaling. Ca^2+^ is an important intracellular second messenger and sensitive to mechanical stimulation. Mechanical stretch increases intracellular Ca^2+ ^concentration by activating Ca^2+^ release from the sarcoplasmic reticulum (SR) and Ca^2+ ^influx [28, 29]. Besides, many researches of splicing regulation have indicated that alternative splicing is controlled by Ca^2+ ^signals [30, 31]. For example, glutamate is the agonist of glutamate receptor, which increases intracellular Ca^2+ ^influx. Under the action of glutamate, ania-6 long mRNA was induced by NMDA receptor [30]. Xie's review summarizes the alternative exons, splicing factors and pre-mRNA elements controlled by Ca^2+ ^signals in response to extracellular stimulation [31]. These studies suggest that alternative splicing in response to mechanical stress might be altered by the communication between Ca^2+ ^signal pathway and splicing factors, splicing elements or splicing machineries. Here, we propose possible mechanisms of the splicing regulation by Ca^2+ ^signals in the cells subjected to mechanical stress. 

The pathways of splicing events regulated by Ca^2+ ^signals in cells subjected to mechanical stress could be divided into two categories: i) Ca^2+ ^signaling pathways coupling with phosphorylation pathways; ii) Ca^2+ ^pathways similar to depolarization. 

There are several researches on the regulation of alternative splicing by depolarization. KCl is a kind of chemical stimulation which causes cellular depolarization and increases the elevation of intracellular calcium. This kind of stimulation decides either inclusion or exclusion of an exon by L-type calciuim channels. *Ania-6* (rattus norvegicus) encodes a truncated form of the cyclin. There are at least two splice variants of *ania-6* gene, *short ania-6* and *long ania-6*. KCl up-regulated the short ania-6 mRNA expression [30]. In rat pituitary GH_3_ cells, 25-50mM KCl decreased the inclusion of stress-axis regulated exon (STREX exon) in transcripts [32]. It is very interesting that Ca^2+ ^influx is induced not only by depolarization but also by mechanical stretching. Ca^2+ ^influx is up-regulated in response to mechanical stimulation by releasing Ca^2+ ^from the sarcoplasmic reticulum (SR) and enhancing L-type Ca^2+ ^currents [28, 29, 33]. On the bases of these results, the alternative variants which are regulated by cellular depolarization may be also regulated by mechanical stress Fig. (**2**).

Alternative splicing is regulated by cis-acting elements and trans-acting factors. The serine/arginine rich protein (SR protein) family and hnRNPs function as essential splicing factors during both constitutive and alternative splicing. Depolarization increased nuclear level of hnRNP A1 in neurons, and changed the alternative splicing form of hnRNP H3 and RNP S1 by affecting the inclusion of their variant exons [34]. Previously mentioned ania-6 whose alternative splicing was regulated by Ca^2+ ^could also be considered as a SR protein. Because its sequences contain arginine/serine-rich domains which are the characteristic of SR splicing factors. It means that Ca^2+ ^also regulates splicing events by modulating the splicing elements and factors. Fig. (**2**) is the possible Ca^2+ ^signal pathway on regulation of alternative splicing by mechanical stress.

### Phosphorylation Signaling Pathway

Phosphorylation of protein plays important roles in alternative splicing regulation. The processes of both spliceosomal assembly and splicing catalysis are regulated by reversible phosporylation. SR proteins are a kind of important splicing factors. Phosphorylation of SR protein in RS domains decides the selection of alternative splicing sites by modulating the sub-cellular localization and activation of SR protein. Phosphokinase and phosphorylation pathways which modulate splicing are also regulated by mechanical stress. The comparison of pathways of splicing regulation and mechanotransduction in cells will show us the mechanisms of splicing regulated by mechanical stress. Fig. (**3**) shows the possible phosphorylation signaling pathway on regulation of alternative splicing by mechanical stress.

* FosB* and *ΔFosB*, the two variants of FosB gene are induced by fluid shear stress at both mRNA and proteins levels *in vivo* and *in vitro* experiments [35]. This regulation depends on ERK activation by the Ca^2+ ^influx through Gd^3+^-sensitive cation channel. *ΔFosB* involves in mechanical stress induced mechanotransduction in cells. Therefore, we propose that splicing is regulated by the pathway of Ca^2+ ^influx-ERK, when cells are subjected to mechanical stimulation.

Nuclear factor kappa B (NF-κB) is one of cis-activating elements of cyclooxygenase2 (*COX-2*) promoter involved in *COX-2* upregulation. Its activation associates with promoting osteoclastogenesis and suppression of collagen synthesis [36]. The expression of *IL-6 *is enhanced by mechanical stretching via the Ca^2+ ^influx- CaMKII-TAK1-SAPKs and NF-κB pathway [37]. *Bcl-x* belongs to the *Bcl-2* family, and has two variants (Bcl-xL and Bcl-xS) generated by splicing. The expression of* Bcl-xL* is reduced by *IL-6* in K562 leukemia cell. We can infer from these evidences that mechanical stimulation regulates alternative splicing by the pathway Ca^2+ ^influx- CaMKII-TAK1-SAPKs and NF-κB-IL-6. 

The activation of Ras is sufficient to regulate *CD45* alternative splicing [38]. The isoform of *CD44* containing v5 exon sequence is induced by the Ras-Raf-MEK-ERK-MAP kinase signaling pathway [39]. Mechanical signals are transported into cells by ECM and integrins on membrane, then these mechanical signals are changed into chemical signals. Focal adhesion kinase (FAK) which links to integrin signals is phosphorylated by its interaction with Src. Further, phosporylated FAK activates ERK pathway [40]. These results indicate that the activation of ERK connects the mechanical stimulation and splicing regulation by the ECM-Integrins-FAK and Src-ERK-MAP kinase pathway. In addition, Rho proteins are also sensitive to mechanical stimuli and transmit mechanical signals [41]. Continuous cyclic mechanical tension (CCMT) reduced the activity of RhoA [42]. Rock which belongs to the family of serine/ threonine kinases is an effector protein of Rho.

RhoA/ROCK signaling pathway plays significant roles in mechanotransduction in force-exposed hPLFs. FAK could be activated by RhoA/ROCK signaling via Integrins-RhoA/Rho-ROCK cascade pathway [43]. After that, the ERK pathway relating to splicing regulation was also activated. So we get another mechanotransduction pathway involved in the regulation of alternative splicing Integrins-RhoA/Rho-ROCK-FAK and Src-ERK-MAP kinase pathway. 

Differential splicing of *CD44* isoforms is up-regulated by Phosphatidylinositol3-kinase (PI3) and PKC pathway [44]. Shear stress activates PI3 kinase in endothelial cells. The activation of PI3 involves in some downstream signaling pathway, including protein kinase B (PKB, also known as Akt) [45]. Recent study also showed that mechanical strain enhanced activation of PI3-kinase/Akt [46]. We suppose that regulation of alternative splicing in response to mechanical stimulation also depends on expression of PI3-kinase.

Protein kinase C (PKC)-dependent pathway plays predominant roles in *CD45* splicing mutants expression [38]. Shear stress induces Ca^2+ ^influx in endothelial cells and subsequently causes activation of PKC/MAPKs pathway [47]. These data combined with splicing regulation pathway of ECM-Integrins-FAK and Src-ERK-MAP, provide us another possible regulation mechanism of alternative splicing Ca^2+ ^influx/PKC/MAPKs. However, it is not sure that if there is any other effector mediating the splicing regulation by PKC.

### Other Pathways

In addition to the above pathways, we find that PI3K signal pathway involves in splicing regulation, too. The study of *VEGF* alternative splicing induced by mechanical stress showed that stretching frequency was related to *VEGF* variants expression [48]. The mRNAs of soluble *VEGF* isoforms (*VEGF_121_, VEGF_165_*) were specifically expressed under low frequency while matrix-bound* VEGF* isoforms (*VEGF_206_, VEGF_189_, VEGF_165_, VEGF_145_*) were expressed under high frequency in human osteoblasts. The mechanism of this process was unclear. However, further research suggested that production of matrix-bound *VEGFs* depended on activation of f-actin polymerisation. Further more, mechanical stimuli could regulate stress fiber formation, which is modulated by the activation of PI3K [49, 50]. It can be speculated that mechanical stress induced activation of PI3K and subsequently active f-actin polymerisation regulated *VEGF* alternative splicing. 

### 4 FUNCTIONS OF MECHANICAL INDUCED SPLICING VARIANTS

Studies have shown that splicing variants induced by mechanical stress could exert feedback mechanisms to the cells during mechanical stretching. 

Mechanical strain generates different effects on *VEGF* alternative splicing. The matrix-bound *VEGF* isoforms which are expressed under high frequency mechanical stress are released by proteases and play roles in metaphyseal vascularization, cartilage resorption and bone formation. Soluble variants which are expressed under low frequency stimulation are important for diffusing of perichondrium, stimulating outgrowth of epiphyseal vascular network and vascular invasion [51].

Alternative splicing isoforms of *TIPs* (*TIP-1* and *TIP-3*) are sensitive to stretch. They make different responses to mechanical stimuli. *TIP-1* is induced by stretching but *TIP-3* is suppressed by stretching. *TIP-1* stimulates myogenesis and *TIP-3* stimulates adipogenesis [52].

* IGF-I* mediates growth and anabolic response in different tissues. *MGF* is one of *IGF-I *splicing isoforms on the basis of its mechanical stimulation. *MGF* is found to be expressed in heart, osteoblast, tendon and brain [53-56]. *MGF* facilitates stress-sensitive tissue regeneration and makes protection of important organs from damaging. After* MGF* is injection, muscle mass increased 20%. Cells treated with *MGF* exhibited high proliferation level [57]. *MGF* also involved in activating satellite cell in the process of muscle repair [58].

Fig. (**4**) describes the process of *IGF-I* alternative splicing induced by mechanical stimulation. Extracellular stress signals enter cells through different membrane receptors and activate series of cascade reaction. Stress signals are changed into chemical signals and transmitted along with mechanotransduction pathway, activating regulators related to alternative splicing. Finally, *IGF-I *pre-mRNA splicing is regulated by mechanical stress and *MGF* is produced by this process. In response to mechanical stress, the 49bp (human) or 52bp (murine) insertion in exon 5 of MGF causes a frame-shift that generates different C-terminal from other IGF-I isoforms. Because *MGF* exhibits special functions on cell proliferation and differentiation, new autocrine *MGF* reacts back to cells which are exposed to mechanical stress.

## 5 PERSPECTIVE

Alternative splicing is a highly coordinated process that relies on a combination of positive and negative-acting factors, intronic and exonic sequence elements and temporal and spatial signal pathways for proper control. Alternative splicing was regulated by many different kinds of extracellular stimulation including mechanical stress, such as mechanical stretching or fluid shear stress. This review mentioned direct and indirect evidences of splicing regulation induced by mechanical signals and speculated possible mechanisms of this regulation process, including Ca^2+ ^signal pathways and phosphorylation signal pathways. Although these results have not been verified by experiments, we present a new direction for alternative splicing study.

Deep understanding of alternative splicing functions and mechanisms has important theoretical and practical significance. First, pre-mRNA splicing has become a novel target for drug design [59]. Second, the immune system utilizes pre-mRNA splicing to expand its gene function [60]. Even the differentiation and self-renewal or pluripotency are regualted by alternative splicing [61]. Numerous immunologically relevant genes undergo alternative splicing [62]. Thus, the study of alternative splicing can reveal a new source of complexity in the immune gene network and help research in disease prevention. Hence, it is important to understand the mechanical signaling system involved in splicing regulation.

## Figures and Tables

**Fig. (1):**
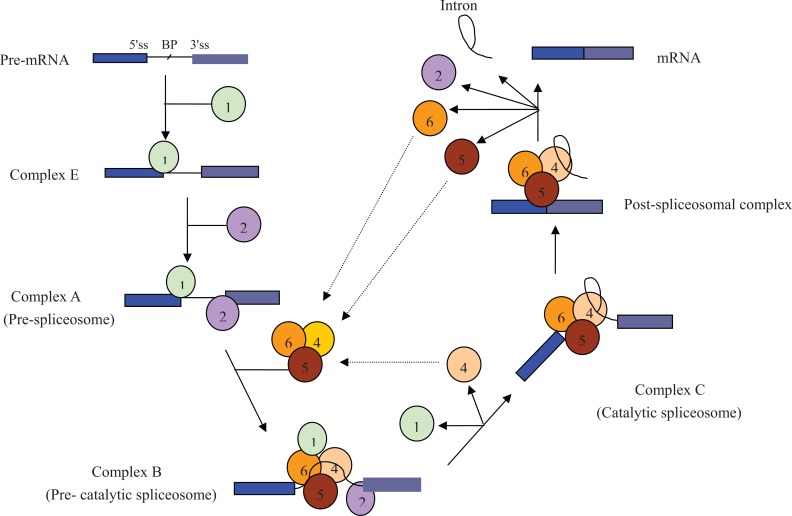
**Pre-mRNA splicing by spliceosome.** ①,②,④,⑤ and ⑥ represent U1snRNP, U2snRNP, U4snRNP, U5snRNP and U6snRNP, respectively. 5'ss represents 5' splice site. BP represents branch point. 3'ss represents 3' splice site.

**Fig. (2):**
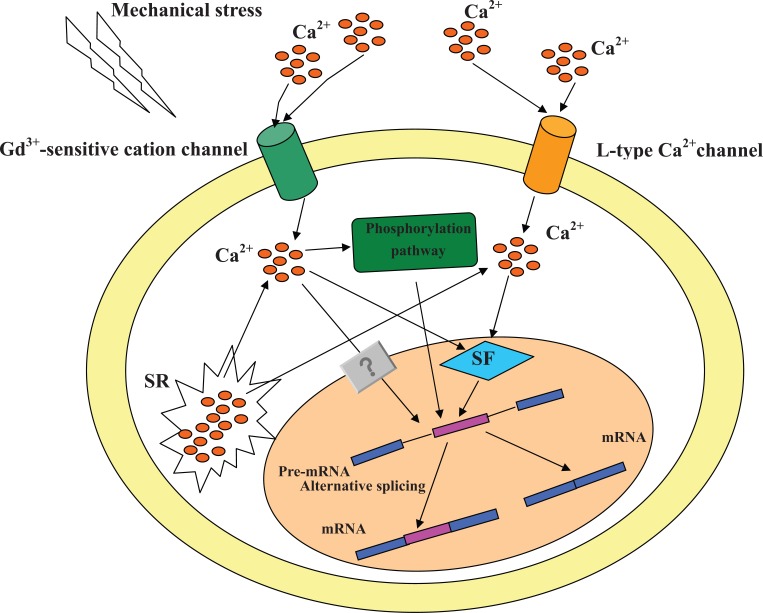
**Ca^2+^ signaling pathway of alternative splicing regulated by mechanical stress.** In response to mechanical stimulation, Ca^2+^ influx is induced by both Gd^3+^-sensitive cation channel and L-type Ca^2+^ channel. Phosphorylation path is activated by Ca^2+^ entried through Gd^3+^- sensitive cation channel (see Fig. **3**), then regulates the expression of different splicing isoforms. Furthermore, the increase of intracellular Ca^2+^ by mechanical stress makes effect on splicing factors, then changes different splicing variants expression.

**Fig. (3):**
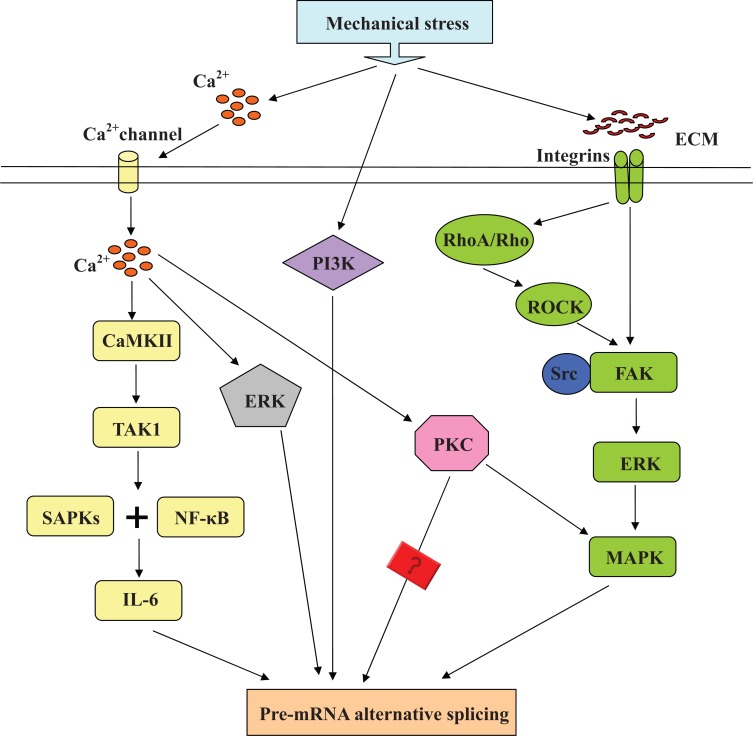
**Phosphorylation signaling pathway of alternative splicing regulated by mechanical stress.** In response to mechanical stimulation, Ca^2+^ influx is induced by Ca^2+^ channel. The pathways of CaMKII-TAK1-SAPKs and NF-κB-IL-6 and PKC are activated by Ca^2+^ influx, then regulate the alternative splicing of pre-mRNA. Integrins which are sensitive to mechanical stimulation, transmit mechanical signals into cells by ECM and activate FAK/ERK/MAPK path way which regulate alternative splicing directly or under help of RhoA/Rho-ROCK signaling. PI3K is another possible signaling that modulates alternative splicing in cells exposed to mechanical stress.

**Fig. (4):**
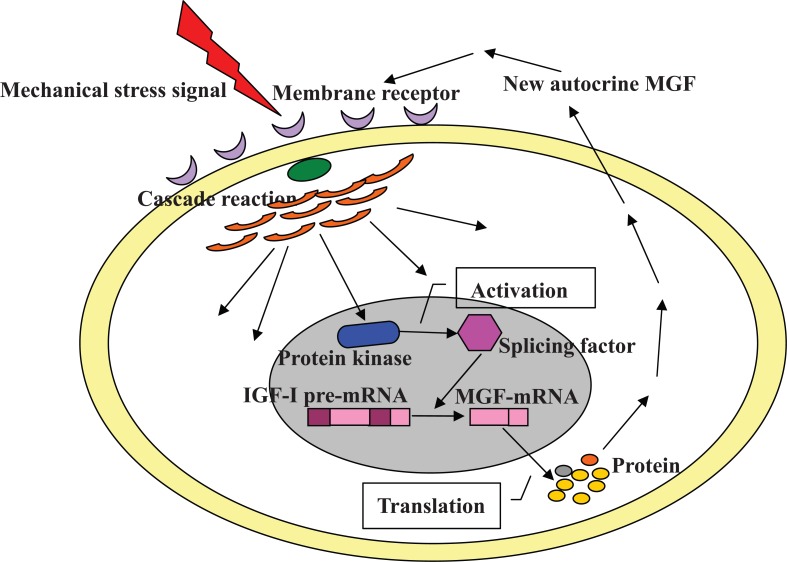
**Description of the process of IGF-I alternative splicing induced by mechanical stimulation.** IGF-I pre-mRNA splicing is regulated by mechanical stress and MGF is produced by this process. Extracellular stress signals enter cells through different membrane receptors and activate series of cascade reaction. Stress signals change into chemical signals and are transmitted along with mechanotransduction pathway, then activate regulation of alternative splicing (such as the phosphorylation of SR factors by protein kinases). Finally, new autocrine MGF reacts back to cells which are exposed to mechanical stress.
